# New Biocompatible Cyanoacrylate and Polylactic Acid Hemostatic Patch: An In Vivo Proof of Concept Study

**DOI:** 10.3390/ma18061271

**Published:** 2025-03-13

**Authors:** Alexandru Ilie-Ene, Victor Petru Tosa, Luciana-Madalina Gherman, Raluca Maria Pop, Lorena-Maria Hantig, Catalin Ovidiu Popa, George Calin Dindelegan

**Affiliations:** 1 Department of Surgery, “Iuliu Hatieganu” University of Medicine and Pharmacy, 8 Victor Babes Street, 400012 Cluj-Napoca, Romania; hantig.lorena.maria@elearn.umfcluj.ro (L.-M.H.); george.dindelegan@umfcluj.ro (G.C.D.); 2 Materials Science and Engineering Department, Technical University of Cluj-Napoca, 103-105 Muncii Ave., 400641 Cluj-Napoca, Romania; catalin.popa@stm.utcluj.ro; 3 Experimental Centre, “Iuliu Haţieganu” University of Medicine and Pharmacy, 6 Louis Pasteur Street, 400349 Cluj-Napoca, Romania; luciana.gherman@umfcluj.ro; 4 Pharmacology, Toxicology and Clinical Pharmacology, Department of Morphofunctional Sciences, “Iuliu Hatieganu” University of Medicine and Pharmacy, 8 Victor Babes Street, 400012 Cluj-Napoca, Romania; raluca.pop@umfcluj.ro

**Keywords:** hemostatic material, topical hemostasis, cyanoacrylate, polylactic acid, experimental liver resection model

## Abstract

Background: Traumatic injuries cause millions of deaths annually, with intra-abdominal hemorrhage as a leading cause. Achieving hemostasis remains challenging. This study evaluates the hemostatic properties of a novel biocompatible cyanoacrylate–polylactic acid patch. Methods: Thirty-six male Wistar rats were randomized into three groups (control, study, and TachoSil^®^) and underwent a standardized liver resection. The hemostasis times were recorded, and blood samples were drawn to assess hemoglobin, hematocrit, and cytokine levels (IL-6 and TNF-α) using validated assays. Results: In the study group, a median time to hemostasis of 94 s was recorded, compared with 256 s for electrocautery (*p* < 0.001) and 120 s for TachoSil^®^ (*p* = 0.010). Hemoglobin levels significantly decreased postoperatively in all groups, with the TachoSil^®^ group showing the lowest median value (from 15.4 g/dL to 11.9 g/dL, *p* = 0.006). Furthermore, only the TachoSil^®^ group exhibited a marked inflammatory response with a significant rise in IL-6 (from 15.6 to 26.8 pg/mL, *p* = 0.04) and TNF-α (from 9.8 to 28.6 pg/mL, *p* = 0.01). Conclusions: The results demonstrated that the novel cyanoacrylate–polylactic acid patch achieved rapid hemostasis and was associated with reduced inflammatory responses compared with both electrocautery and TachoSil^®^. These findings suggest that this hybrid material may offer a safer and more effective hemostatic alternative.

## 1. Introduction

Injuries, encompassing both unintentional incidents and those related to violence, result in the deaths of 4.4 million individuals globally each year, accounting for nearly 8% of all fatalities [1]. Intra-abdominal hemorrhage is a leading cause of death in trauma patients, with the abdomen being the most common site of bleeding (up to 44.3% of cases) [2]. Patients with abdominal trauma frequently sustain parenchymal organ damage, notably to the liver and spleen [3]. The wide variety of bleeding control methods, ranging from simple compression to complex technologies, reflects the absence of a single, universally effective solution [4].

Out of the outstanding plethora of hemostatic materials already described, cyanoacrylate-based compounds exhibit various advantageous properties [5]. Cyanoacrylate (CA) is a synthetic liquid monomer that rapidly polymerizes when in contact with weak bases (such as water or alcohol), forming stable bounds. CA are adhesives approved for clinical use by the FDA [6]. While earlier CA formulations were toxic due to harmful degradation byproducts, modern CA compounds have been developed and shown to be biocompatible [7,8,9]. CA adhesives have a variety of medical applications, including skin closure, hernia mesh fixation, endoscopic obliteration of esophageal or gastric varices, and arterial embolization [10,11,12,13,14]. Experimental studies have explored their use in vascular or intestinal anastomosis [15,16]. And more importantly for this study, CA demonstrated good hemostatic properties [5,17]. CA is a highly effective mechanical hemostat. It significantly outperformed thromboplastin in clotting anticoagulated blood and demonstrated in vivo hemostatic efficacy comparable to established agents, suggesting potential clinical utility [18]. In a prospective randomized study, Martines et al. concluded that the application of CA on top of standard hemostatic techniques effectively prevents delayed bleeding after endoscopic submucosal dissection of precancerous lesions or early stage gastrointestinal cancer, with a statistically significant reduction in postoperative bleeding rates [19]. CA closure of surgical wounds after impacted wisdom tooth removal resulted in significantly less bleeding on the first day post-surgery [20]. Independent prospective randomized controlled studies by Suthar et al. and Narsingyani et al. on alveoloplasty wounds showed that CA was significantly faster for hemostasis than sutures, with mean time differences of 2.26 min (*p* < 0.001) and 132.92 ± 4.97 s (*p* < 0.001), respectively [21,22].

This experimental study aimed to validate a novel biocompatible hemostatic patch utilizing CA and polylactic acid (PLA) [23]. PLA was chosen as a resorbable and biocompatible support matrix [24]. An uncontrolled hemorrhage model (liver resection in rats) was employed to compare the hemostatic effectiveness of our CA–PLA patch against electrocautery and TachoSil^®^ [25]. The study analyzed bleeding time, total blood loss, and inflammatory response.

## 2. Materials and Methods

### 2.1. Development Process of the New Hemostatic Material

A nonwoven membrane was produced via electrospinning using PLA powder (average Mw ≈ 60,000 g/mol, Sigma Aldrich, St. Louis, MO, USA). The resulting substrate was composed of intertwined fibers with a diameter ranging from 2.8 to 10.7 μm (mean diameter of 7.24 μm). Patches were cut out measuring 3 by 1.5 cm to optimize the intraoperative application process. Using a 3D printer—Crealty Ender 5 (Shenzhen Creality 3D Technology Co., Ltd., Shenzhen, China), a protective box was fashioned out of PLA to accommodate the hemostatic patches. The patches were introduced in boxes (1 patch per box), which in turn were placed in a sterile vacuum bag that was fitted with a silicone sleeve seal. The PLA patches were inoculated with n-hexyl cyanoacrylate glue (Ifabond™—Peters Surgical, Boulogne-Billancourt Cedex, France) by injection with a syringe through the silicone seal. Prior to this step, the sterile bag was vacuum-sealed, and then the PLA patches were washed (through the silicone sleeve seal) using sulfur dioxide (SO_2_) gas, generated by the reaction between hydrochloric acid (HCl) and sodium sulfite (Na_2_SO_3_). This was a crucial step in production that ensured the inhibition of CA polymerization once it was introduced. The inoculation process took place inside a glovebox filled with nitrogen gas and using silica gel to ensure inert and moisture-free conditions. After completing the defined steps, approximately 0.1 mL of tissue adhesive was applied to each PLA substrate (150 mm^2^ area) within the sealed bag, resulting in a density of approximately 0.67 mL/cm^2^.

**Figure 1:**
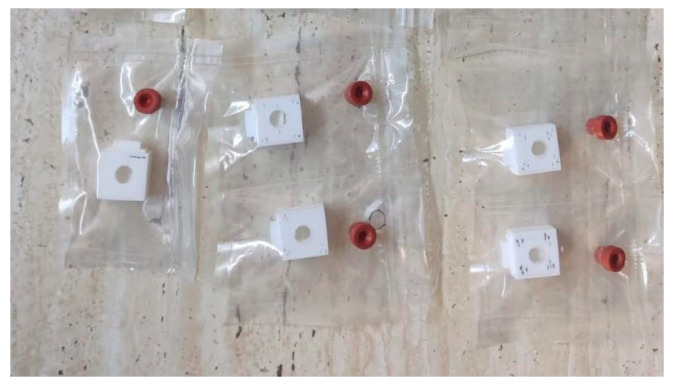
Individually packed, vacuum-sealed CA–PLA patches.

Characteristics and functional properties of the PLA–cyanoacrylate patches have been described in another article [23]. The vacuum-sealed CA–PLA patches were individually packed, as described in Figure 1.

### 2.2. Hemostasis Experimental Model

**Figure 2:**
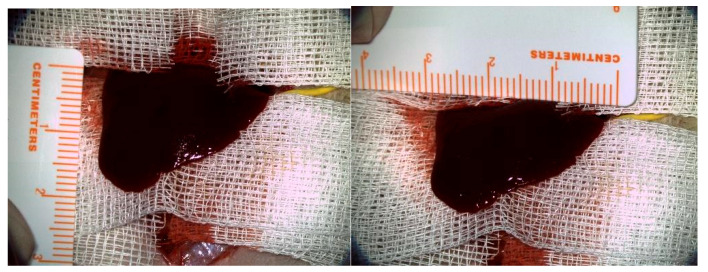
Rats’ left hepatic lobe exposed and measured pre-resection.

The experimental protocol involved a liver laceration model, previously characterized and validated for its consistent induction of uncontrolled hemorrhage in rats [25]. Thirty-six male Wistar rats (weighing approximately 350 g) were used in this study. Prior to the laparotomy, the rats were weighed, and the appropriate dose of anesthesia, 2 parts Ketamine (Vetased, Biotur Exim SRL, Alexandria, Romania) for 1 part Xylazine (Xylazin, Biotur Exim SRL, Alexandria, Romania), was intramuscularly administered; afterwards, the abdomen was shaved, and the preparation of the operating field was completed using a povidone-iodine solution and sterile fields. A xipho-subumbilical midline laparotomy was performed, and abdominal wall retractors were placed. The left lobe of the liver was exposed and isolated. A 3 × 1.5 cm segment was measured and resected as shown in Figure 2.

Liver hemostasis was achieved using one of the following three methods: bipolar electrocautery (control group, *n* = 6), the novel CA–PLA hemostatic patch (study group, *n* = 15), and finally a fibrinogen/thrombin patch—TachoSil^®^—Corza Medical Gmbh., Jestetten, Germany (TachoSil^®^ Group, *n* = 15), as presented in Table 1.

**Figure 3:**
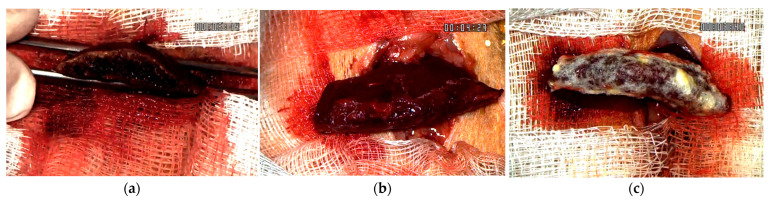
Final aspect after achieving hemostasis: (**a**) bipolar electrocautery; (**b**) novel CA–PLA patch; (**c**) TachoSil^®^.

Intraoperative footage regarding the hemostasis process can be consulted in the Supplementary Materials Section (Videos S1–S3). The final aspect of the resection plane for all 3 hemostatic methods after achieving hemostasis is described in Figure 3.

After hemostasis was achieved, abdominal wall closure was performed using 4-0 polydioxanone sutures in a continuous double-layer fashion. The rats were kept under observation for 24 h before returning to their original housing cages where water and food were available ad libitum.

### 2.3. In Vivo Testing of the New PLA–Cyanoacrylate Hemostatic Material

The time needed to obtain total hemostasis was recorded (time to hemostasis—TTH), starting from the moment when the liver segment was resected until there was no blood oozing from the resection plane.

To minimize blood sampling while capturing the expected peak changes in blood counts on the first postoperative day, we collected samples at three time points: preoperative, day one, and day three. We chose this methodology to reduce the trauma of repeated blood sampling and to limit the financial burden of excessive testing. Total blood loss was calculated by comparing preoperative (T0) hemoglobin (Hb) levels with those in postoperative day one (T1). A complete blood count was performed from one milliliter of venous blood (drawn from the retro orbital plexus) of each specimen prior to the surgical intervention and another one in the 1st postoperative day. The difference between the two measurements was interpreted as total blood loss.

An inflammatory reaction assessment was performed by comparing leukocytes, tumor necrosis factor alpha (TNF-α), and interleukin-6 (IL-6) levels preoperatively with those in the 3rd postoperative day. For TNF-alpha and IL-6, the enzyme-linked immunosorbent assay (ELISA) technique was used on venous blood drawn in the same fashion as for the complete blood count.

The animal part of the experiment was approved by the Romanian ANSVSA—Sanitary Veterinary and Food Safety Department of Cluj through the project authorization No. 377/25.08.2023.

### 2.4. Statistical Analysis

All data were tested for normality using the Shapiro–Wilk test. If the distribution of a given variable was approximately normal, parametric methods were applied: one-way ANOVA for between-group comparisons (C vs. S vs. T) and paired *t*-tests for within-group pre–post changes. Tukey’s test were employed for post-hoc pairwise analyses when the ANOVA indicated significant differences. When data were nonparametric, we employed the Kruskal–Wallis test for three-group comparisons and the Mann–Whitney U test for pairwise group analyses. Within-group changes were assessed by the Wilcoxon signed-rank test if data were not normally distributed. A *p*-value < 0.05 was considered statistically significant. Given concerns with the distribution of certain parameters and for clarity, we present the main outcomes as median and interquartile range (IQR). All statistical procedures were performed in SPSS v.25 (IBM Corp., Armonk, NY, USA).

## 3. Results

The median TTH for the C group was 256 s (IQR: 245–295), whereas the S group achieved hemostasis in just 94 s (IQR: 86–98). The T group had a median TTH of 120 s (IQR: 78–131). Statistically significant differences were noted when comparing these groups. Specifically, the S group’s TTH was significantly lower than that of the C group (*p* < 0.001) and significantly lower than the T group (*p* = 0.010), as presented in Table 2.

In the C group, Hb levels decreased from a median of 16.1 g/dL (IQR: 15.5–17.0) preoperatively to 12.4 g/dL (IQR: 11.7–12.7) postoperatively. Similarly, the S group’s median Hb dropped from 16.0 g/dL (IQR: 14.7–17.8) to 12.8 g/dL (IQR: 11.4–13.5), and the T group decreased from 15.4 g/dL (IQR: 14.5–16.6) to 11.9 g/dL (IQR: 10.7–12.4). All groups experienced statistically significant reductions in Hb, with *p*-values of 0.004 in the C group, 0.002 in the S group, and 0.006 in the T group (Table 3).

The C group’s median hematocrit (Ht) decreased from 44.0% (IQR: 42.0–46.0) to 34.5% (IQR: 33.2–35.5%), and the S group’s Ht declined from 45.8% (IQR: 44.2–48.1) to 35.0% (IQR: 33.5–36.3%). The T group’s Ht fell from 43.9% (IQR: 41.0–47.1) to 32.7% (IQR: 30.6–35.1%). These reductions were statistically significant, with *p*-values of 0.013 for the C group, <0.001 for the S group, and 0.007 for the T group (Table 4).

The median leukocyte counts for the C group changed modestly from 9.5 × 10^9^/L (IQR: 6.9–13.0) to 9.8 × 10^9^/L (IQR: 7.2–11.8), while the S group’s counts shifted from 12.1 × 10^9^/L (IQR: 9.4–16.0) to 10.5 × 10^9^/L (IQR: 8.3–13.2). The T group’s counts moved from 11.8 × 10^9^/L (IQR: 8.2–14.1) to 10.8 × 10^9^/L (IQR: 8.5–15.9). No statistically significant differences were detected in leukocyte counts among the groups, and therefore, only numerical trends were observed, as in Table 5.

In the T group, IL-6 levels increased significantly from a median of 15.6 pg/mL (IQR: 9.4–20.9) preoperatively to 26.8 pg/mL (IQR: 12.9–40.5) postoperatively (*p* = 0.04). Although the C and S groups showed numerical increases or near-constant levels, these changes did not reach statistical significance. Thus, the significant elevation in IL-6 observed in the T group pointed to an enhanced inflammatory response associated with the fibrinogen/thrombin patch compared with the other hemostatic techniques (Table 6).

The T group also exhibited a significant increase in TNF-α levels, with median values rising from 9.8 pg/mL (IQR: 0.9–5.2) preoperatively to 28.6 pg/mL (IQR: 5.8–60.3) postoperatively (*p* = 0.01). In contrast, the C and S groups did not show significant changes in TNF-α. This significant increase in TNF-α further supported the observation that the TachoSil^®^ patch was associated with a stronger inflammatory response (Table 7).

**Figure 4:**
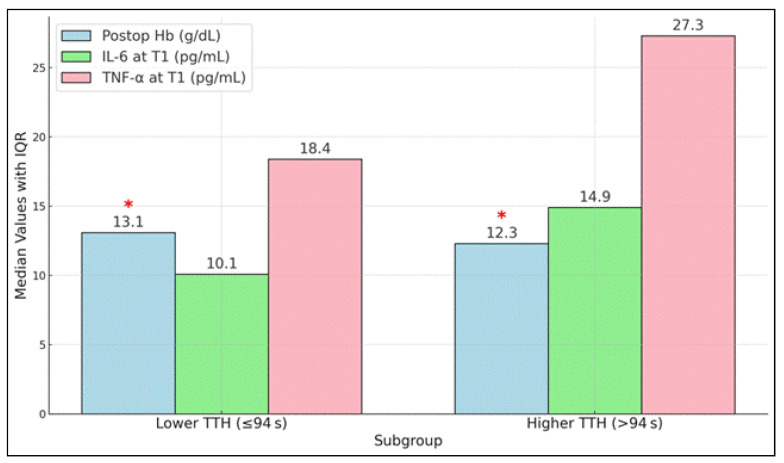
Subgroup analysis in the study group (novel adhesive) by lower (≤median) vs. higher (>median) time to hemostasis. “*” marks the statistically significant difference between subgroups.

Within the S group, rats with a lower time to hemostasis (TTH ≤ 94 s; *n* = 8) achieved hemostasis in a range of 67–94 s and maintained a higher postoperative Hb level of 13.1 g/dL (IQR: 12.4–13.5). In contrast, those with a higher TTH (>94 s; *n* = 7) had a hemostasis range of 95–112 s and a lower postoperative Hb of 12.3 g/dL (IQR: 11.0–12.9). The difference in postoperative Hb between the lower and higher TTH subgroups was statistically significant (*p* = 0.04), indicating that faster hemostasis was correlated with better preservation of Hb levels in the S group (Table 8 and Figure 4).

**Figure 5:**
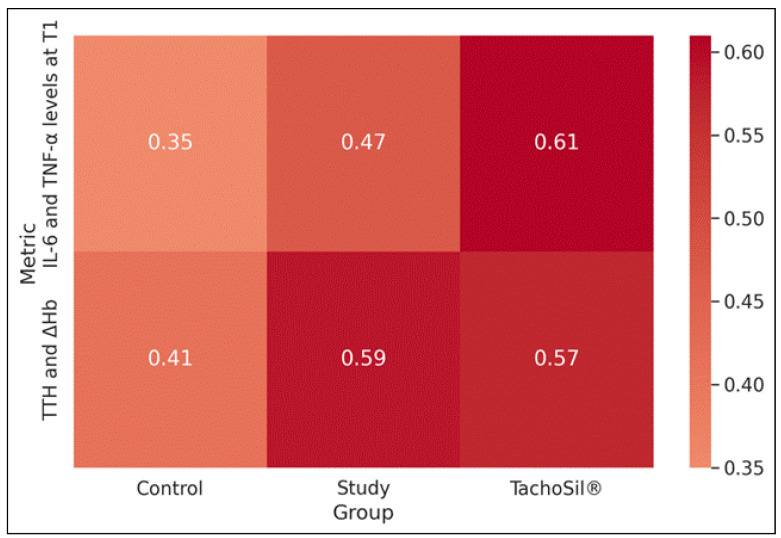
Correlation matrix.

In the T group, a strong positive correlation was found between IL-6 and TNF-α levels at T1 (ρ = 0.61, *p* = 0.01). Additionally, significant positive correlations between time to hemostasis (TTH) and the reduction in Hb (ΔHb) were observed in the S group (ρ = 0.59, *p* = 0.01) and the T group (ρ = 0.57, *p* = 0.02). These significant correlations underscored that prolonged hemostasis was associated with greater blood loss and that, particularly in the T group, higher cytokine levels were interrelated (Table 9 and Figure 5).

**Figure 6:**
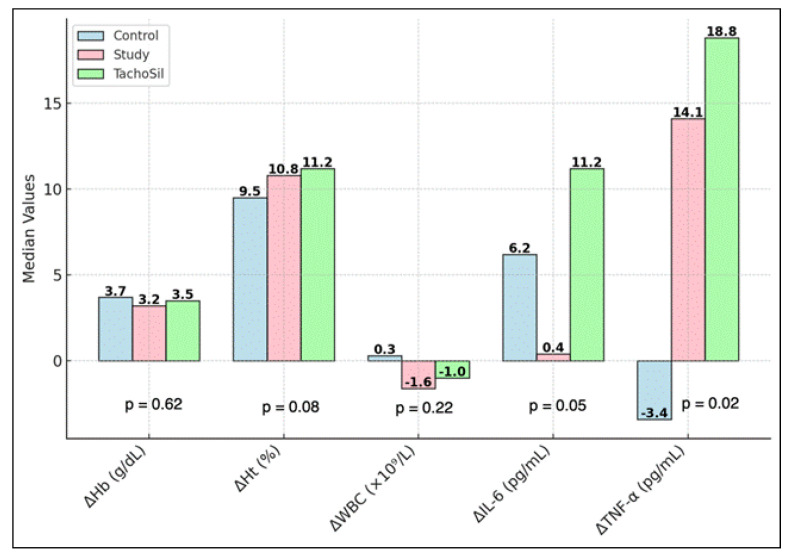
Inter-group comparisons of changes (Δ) in parameters and TTH. *p* < 0.05 represents statistical significance.

Inter-group comparisons revealed statistically significant differences in several parameters. The change in IL-6 (ΔIL-6) was significantly different among groups (*p* = 0.05), and the change in TNF-α (ΔTNF-α) was significantly higher in the T group (+18.8 pg/mL, IQR: 8.0–40.0) compared with the C group (−3.4 pg/mL, IQR: −8.0 ± 2.0) and S group (+14.1 pg/mL, IQR: 5.0–30.0) (*p* = 0.02). Most notably, the TTH demonstrated a highly significant divergence between groups (*p* < 0.001), with the S group demonstrating the shortest median TTH of 94 s (IQR: 86–98) compared to 256 s (IQR: 245–295) in the C group and 120 s (IQR: 78–131) in the T group, as presented in Table 10 and Figure 6.

## 4. Discussion

There are several experimental studies reporting on the hemostatic properties of CA compounds. Using a novel airflow-directed in situ electrospinning method, Jiang et al. created a nonwoven CA web directly on the wound site. These ultrathin polymer fibers rapidly halt superficial bleeding, forming a robust barrier within seconds [26]. An improved in situ electrospinning technique, incorporating an electric field modifier for more precise and controlled CA fiber deposition, was developed. This refined method achieved better hemostasis compared to the airflow-assisted control [27]. The same research team, in a subsequent study, used a rat partial nephrectomy model to test their portable, handheld auxiliary electrode for precise, in situ CA application. They observed faster hemostasis compared to standard CA spraying and greater precision than the airflow-assisted method [28]. Gao et al. showed that a novel hemostatic plug (composed of PLA, gelatin, and absorbable hemostatic particles), secured with an in situ electrospun CA film, effectively stopped bleeding in a cardiac (left ventricle wall) perforation model using minipigs, rabbits, and rats. This technique outperformed traditional medical gauze in achieving and maintaining hemostasis, even under high pressure [29].

The hemostatic properties of the new CA–PLA patches have been demonstrated in this study. The new material achieved total hemostasis significantly faster when compared to the bipolar electrocautery and with one of the most performant hemostatic materials available on the market (TachoSil^®^). The amount of blood lost proved to be a constant indifferent to the hemostatic method used. Our results indicate that, in the CA–PLA patch group, faster hemostasis is correlated with higher hemoglobin levels on the first postoperative day.

Regarding the inflammatory reaction to the hemostatic method employed, it seems that the new CA–PLA patch is truly biocompatible, a fact proven by the lack of foreign-body reaction shown in the subjects of the S group. Inflammation markers used in this study (leucocytes, IL-6, and TNF-alpha) have shown similar levels when comparing the new hemostatic material with the C group. These results are in contrast with the ones observed in the T group, which showed a significant increase in inflammation markers and a positive correlation between the time until hemostasis, amount of blood loss, and the severity of inflammation. The absence of inflammatory response to the CA–PLA patch in this study might be credited to the fact that only a small amount of CA was used to impregnate the electrospun PLA substrate, and thus trace amounts of toxic degradation byproducts were produced.

The biocompatibility of the CA–PLA complex is based on the inherent property of the two components’ ability of being absorbed in the human body [7,30,31]. Even though degradation byproducts of the CA are toxic, there are newer polymers recently designed that proved to be nontoxic and suitable for safe intracorporeal use [9,32]. PLA is a biopolymer widely used in medicine as scaffold material for different types of cells, or as drug delivery systems, microcapsules, microspheres and thin coatings; or in surgical sutures and meshes and even for bone fixation (surgical screws, pins, rods, and plates) [24,33]. Hydrolytic degradation is the mechanism by which PLA is broken down and absorbed in the body [34].

A theoretical concern is that the CA constituent of the patch may promote more extensive peritoneal adhesion development relative to other hemostatic agents [17]. Alongside proving its resorbability potential (both CA and PLA are individually proven resorbable materials), the matter of foreign-body reaction/peritoneal adhesions formation should be the aim of further research.

A key advantage of the new CA–PLA hemostatic patch is its 100% synthetic composition. By excluding biological components, including human thrombin and bovine collagen, the patch avoids the potential immunologic and epidemiologic risks observed in isolated instances with hemostatic agents like TachoSil^®^ [35,36]. A further benefit of the CA–PLA patch’s synthetic composition is its suitability for efficient and economical mass production.

The application process of the new hemostatic patch is safer due to the fact that it can be repositioned or even removed with ease if placed incorrectly. This feature is possible because the CA takes approximately 30–60 s in order to completely polymerize and form its strong adhesive bonds [37]. The application of medical-grade CA, confined to droplet pens and particle pulverizers, is inherently uncontrolled, leading to imprecise deposition and precluding any readjustment or removal in the event of erroneous application [5].

This study, while demonstrating promising results for the novel CA–PLA hemostatic patch, is subject to certain limitations. Firstly, the study’s evaluation of the patch’s efficacy was restricted to a single hemorrhagic wound model. Specifically, the type of wound included in the study does not fully represent the diverse range of clinical scenarios where hemostatic agents are required. Expanding the scope of future investigations to encompass a wider variety of wound characteristics, such as different tissue types, severity levels, and bleeding mechanisms, would provide a more comprehensive understanding of the patch’s applicability. Secondly, the in vivo fate of the CA–PLA patch material was not thoroughly investigated. This includes aspects such as the patch’s degradation rate, biocompatibility over extended periods, and potential for tissue integration or adverse reactions. Understanding the long-term behavior of the patch within the body is crucial for assessing its safety and efficacy in clinical practice. By addressing these limitations, future studies can provide a more robust and complete evaluation of the CA–PLA hemostatic patch’s potential for clinical translation.

## 5. Conclusions

The results of this study demonstrated that the novel CA–PLA hemostatic patch exhibited significant advantages in achieving rapid hemostasis and minimizing inflammation when compared to two commonly used hemostatic methods: electrocautery and TachoSil^®^. Despite promising results, this study’s limited wound model and lack of in vivo fate investigation necessitate broader future research for comprehensive clinical validation of the CA–PLA patch. These findings collectively point towards the promising potential of this hybrid CA–PLA material as a safer and more effective hemostatic agent. The combination of rapid hemostasis and reduced inflammation addresses key clinical needs and highlights the material’s potential to improve patient outcomes.

## Figures and Tables

**Table 1:** Group allocation and sample sizes.

Group	Hemostatic Method	Number of Rats (n)
Control (C)	Electrocautery	6
Study (S)	Novel CA–PLA patch	15
TachoSil^®^ (T)	Fibrinogen/Thrombin patch (TachoSil^®^)	15

PLA—polylactic acid.

**Table 2:** Time to hemostasis in seconds.

Group	n	TTH, Median (IQR)	*p*-Value (Kruskal–Wallis)
Control	6	256 (245–295)	–
Study	15	94 (86–98)	<0.001 vs. C; 0.010 vs. TachoSil^®^
TachoSil^®^	15	120 (78–131)	<0.001 vs. C

TTH—time to hemostasis; IQR—interquartile range.

**Table 3:** Hemoglobin (Hb, g/dL) changes: preoperative (T0) vs. postoperative (T1).

Group	Time Point	Hb, Median (IQR)	*p*-Value (Wilcoxon)
Control	T0	16.1 (15.5–17.0)	–
	T1	12.4 (11.7–12.7)	0.004
Study	T0	16.0 (14.7–17.8)	–
	T1	12.8 (11.4–13.5)	0.002
TachoSil^®^	T0	15.4 (14.5–16.6)	–
	T1	11.9 (10.7–12.4)	0.006

Hb—hemoglobin; IQR—interquartile range.

**Table 4:** Ht, % changes: preoperative (T0) vs. postoperative day 1 (T1) [Median (IQR)].

Group	Time Point	Ht, Median (IQR)	*p*-Value (Wilcoxon)
C	T0	44.0 (42.0–46.0)	–
	T1	34.5 (33.2–35.5)	0.013
Study	T0	45.8 (44.2–48.1)	–
	T1	35.0 (33.5–36.3)	<0.001
TachoSil^®^	T0	43.9 (41.0–47.1)	–
	T1	32.7 (30.6–35.1)	0.007

Ht—hematocrit; IQR—interquartile range.

**Table 5:** Leukocyte (WBC, ×10^9^/L) counts: preoperative (T0) vs. postoperative day 1 (T1).

Group	Time Point	WBC, Median (IQR)	*p*-Value (Wilcoxon)
Control	T0	9.5 (6.9–13.0)	–
	T1	9.8 (7.2–11.8)	0.6
Study	T0	12.1 (9.4–16.0)	–
	T1	10.5 (8.3–13.2)	0.18
TachoSil^®^	T0	11.8 (8.2–14.1)	–
	T1	10.8 (8.5–15.9)	0.3

WBC—white blood cells; IQR—interquartile range.

**Table 6:** IL-6 (pg/mL) preoperative (T0) vs. postoperative day 3 (T1).

Group	Time Point	IL-6, Median (IQR)	*p*-Value (Wilcoxon)
Control	T0	10.7 (5.9–15.3)	–
	T1	16.9 (8.0–21.9)	0.1
Study	T0	11.7 (4.0–20.1)	–
	T1	12.1 (4.3–28.2)	0.83
TachoSil^®^	T0	15.6 (9.4–20.9)	–
	T1	26.8 (12.9–40.5)	0.04

IL—interleukin; IQR—interquartile range.

**Table 7:** TNF-α (pg/mL) preoperative (T0) vs. postoperative day 3 (T1).

Group	Time Point	TNF-α, Median (IQR)	*p*-Value (Wilcoxon)
Control	T0	16.7 (5.6–29.5)	–
	T1	13.3 (5.0–20.2)	0.55
Study	T0	8.8 (0.0–15.3)	–
	T1	22.9 (5.5–40.0)	0.18
TachoSil^®^	T0	9.8 (0.9–5.2)	–
	T1	28.6 (5.8–60.3)	0.01

TNF—tumor necrosis factor; IQR—interquartile range.

**Table 8:** Subgroup analysis in the study group (novel adhesive) by lower (≤median) vs. higher (>median) time to hemostasis.

Subgroup (Study)	n	TTH Range (s)	Postop Hb, Median (IQR)	IL-6 at T1, Median (IQR)	TNF-α at T1, Median (IQR)
Lower TTH (≤94 s)	8	67–94	13.1 (12.4–13.5)	10.1 (3.3–20.5)	18.4 (2.9–30.0)
Higher TTH (>94 s)	7	95–112	12.3 (11.0–12.9)	14.9 (5.1–35.6)	27.3 (8.6–42.1)
*p*-value (Mann–Whitney)	–	–	0.04	0.29	0.2

TTH—time to hemostasis; IQR—interquartile range; TNF—tumor necrosis factor; IL—interleukin; Hb—hemoglobin.

**Table 9:** Correlation between IL-6 and TNF-α levels at T1 and time to hemostasis with ΔHb (Preoperative–Postoperative) across all groups.

Correlations	Group	ρ (rho)	*p*-Value
IL-6 and TNF-α levels at T1			
	Control	0.35	0.58
	Study	0.47	0.09
	TachoSil^®^	0.61	0.01
TTH and ΔHb			
	Control	0.41	0.42
	Study	0.59	0.01
	TachoSil^®^	0.57	0.02

TNF—tumor necrosis factor; TTH—time to hemostasis.

**Table 10:** Inter-group comparisons of changes (Δ) in parameters and TTH.

Parameter	Control (*n* = 6) Median (IQR)	Study (*n* = 15) Median (IQR)	TachoSil^®^ (*n* = 15) Median (IQR)	*p*-Value
ΔHb (g/dL)	3.7 (3.0–4.3)	3.2 (2.6–4.6)	3.5 (2.8–4.7)	0.62
ΔHt (%)	9.5 (7.0–12.0)	10.8 (8.2–12.8)	11.2 (9.0–13.2)	0.08
ΔWBC (×10^9^/L)	+0.3 (−0.5–1.2)	−1.6 (−2.0–−0.8)	−1.0 (−2.5–1.0)	0.22
ΔIL-6 (pg/mL)	+6.2 (+2.1 ± 10.0)	+0.4 (−2.0 ± 5.0)	+11.2 (+4.0 ± 25.0)	0.05
ΔTNF-α (pg/mL)	−3.4 (−8.0 ± 2.0)	+14.1 (+5.0 ± 30.0)	+18.8 (+8.0 ± 40.0)	0.02
TTH (s)	256 (245–295)	94 (86–98)	120 (78–131)	<0.001

TNF—tumor necrosis factor; IQR—interquartile range; TTH—time to hemostasis.

## Data Availability

The original contributions presented in this study are included in the article/Supplementary Materials. Further inquiries can be directed to the corresponding author.

## Supplementary Materials

The following supporting information can be downloaded at https://zenodo.org/records/14875155?token=eyJhbGciOiJIUzUxMiJ9.eyJpZCI6IjFlNTRkYzAyLTZlNjAtNDZkZi1hNzA4LTUyY2QwM2NmZjE0MCIsImRhdGEiOnt9LCJyYW5kb20iOiJjYzJkNzAyYmUyNjNmOGVkOWFiMWQwMDEyMmY2MDg5MiJ9.4856KoEKA2v62rpk1eUIsy8MFJ2Z9FsWUnN3_BlGS2nfxmnmkeZw31WZx1Ya2QeXhh2phSmQATwvg9JoHdQC5w (accessed on 15 February 2025), Video S1: bipolar electrocautery—demo; Video S2: CA–PLA hemostatic patch—demo; Video S3: TachoSil—demo.

## Abbreviations

The following abbreviations are used in this manuscript:

CA	Cyanoacrylate
PLA	Polylactic acid
C	Control
S	Study
T	TachoSil^®^
TTH	Time to hemostasis
Hb	Hemoglobin
T0	Preoperative
T1	Postoperative
TNF-α	Tumor necrosis factor alpha
IL-6	Interleukin-6
ELISA	Enzyme-linked immunosorbent assay
IQR	Interquartile range
